# Comparative chloroplast genomics and phylogenetics of *Fagopyrum esculentum *ssp. *ancestrale *– A wild ancestor of cultivated buckwheat

**DOI:** 10.1186/1471-2229-8-59

**Published:** 2008-05-20

**Authors:** Maria D Logacheva, Tahir H Samigullin, Amit Dhingra, Aleksey A Penin

**Affiliations:** 1 Faculty of Bioengineering and Bioinformatics, M.V. Lomonosov Moscow State University, Moscow, Russia; 2 Department of Evolutionary Biochemistry, A.N. Belozersky Institute, M.V. Lomonosov Moscow State University, Moscow, Russia; 3 Department of Horticulture and Landscape Architecture, Washington State University, Pullman, USA; 4 Department of Genetics, Biological Faculty, M.V. Lomonosov Moscow State University, Moscow, Russia

## Abstract

**Background:**

Chloroplast genome sequences are extremely informative about species-interrelationships owing to its non-meiotic and often uniparental inheritance over generations. The subject of our study, *Fagopyrum esculentum*, is a member of the family Polygonaceae belonging to the order Caryophyllales. An uncertainty remains regarding the affinity of Caryophyllales and the asterids that could be due to undersampling of the taxa. With that background, having access to the complete chloroplast genome sequence for *Fagopyrum *becomes quite pertinent.

**Results:**

We report the complete chloroplast genome sequence of a wild ancestor of cultivated buckwheat, *Fagopyrum esculentum *ssp. *ancestrale*. The sequence was rapidly determined using a previously described approach that utilized a PCR-based method and employed universal primers, designed on the scaffold of multiple sequence alignment of chloroplast genomes. The gene content and order in buckwheat chloroplast genome is similar to *Spinacia oleracea*. However, some unique structural differences exist: the presence of an intron in the *rpl2 *gene, a frameshift mutation in the *rpl23 *gene and extension of the inverted repeat region to include the *ycf1 *gene. Phylogenetic analysis of 61 protein-coding gene sequences from 44 complete plastid genomes provided strong support for the sister relationships of Caryophyllales (including Polygonaceae) to asterids. Further, our analysis also provided support for *Amborella *as sister to all other angiosperms, but interestingly, in the bayesian phylogeny inference based on first two codon positions *Amborella *united with Nymphaeales.

**Conclusion:**

Comparative genomics analyses revealed that the *Fagopyrum *chloroplast genome harbors the characteristic gene content and organization as has been described for several other chloroplast genomes. However, it has some unique structural features distinct from previously reported complete chloroplast genome sequences. Phylogenetic analysis of the dataset, including this new sequence from non-core Caryophyllales supports the sister relationship between Caryophyllales and asterids.

## Background

Chloroplasts are hypothesized to have evolved from ancient endosymbiotic cyanobacteria. They are semi-autonomous possessing their own genome that codes for a set of proteins, which orchestrate the process of photosynthesis and other house-keeping functions. The non-meiotic and mostly uniparental inheritance of chloroplast genes render them as most informational entities in plant phylogenetic studies. Technological enhancements and consequent reduction of time in sequence capture have enabled sequencing of several chloroplast genomes recently. The discipline of plant phylogenetics has been the largest beneficiary of these technological advances. The phylogenetic trees derived from the analysis of whole genome sequences are completely or near-completely resolved, with highly supported nodes. Further, analysis of chloroplast gene-evolution rates can be informative about nodal support as recently demonstrated in Saxifragales where slow evolving genes from the chloroplast inverted repeat region provided support for deep level divergences [1]. Despite the availability of these datasets, complete chloroplast genome sequence-based phylogenies are prone to artifacts caused by incomplete taxon sampling [2-4]. Therefore, availability of complete chloroplast genome sequences from additional taxa is highly desirable for robust phylogenetic studies.

This study reports the complete chloroplast genome sequence from *Fagopyrum esculentum *ssp. *ancestrale*, a wild ancestor of cultivated buckwheat [5]. This species belongs to the family Polygonaceae. According to APGII [6] Polygonaceae is a member of the order Caryophyllales; however this family represents a separate group within it, the so called non-core Caryophyllales [7] and sometimes it is treated as a separate order, Polygonales [8]. Both phylogenetic and genomic studies are lacking for this group. In addition, the affinities of the order Caryophyllales as a whole also remain debatable. All chloroplast genome sequence-based phylogenies obtained till date place *Spinacia *(the only representative of Caryophyllales with a known chloroplast genome sequence) as sister to asterids (for example see [9,10]). Other studies incorporating lesser number of genes but broader taxon sampling placed them at the base of the clade which includes asterids and rosids [6,11]. To validate if the sister relationship of Caryophyllales and asterids is due to taxon undersampling in Caryophyllales, additional sequence information is highly desirable. Therefore, inclusion of the buckwheat chloroplast genome sequence in a comprehensive phylogenetic analysis is expected to aid in addressing the affinity issue of the Caryophyllales.

The sequence of *Fagopyrum *chloroplast genome, besides its phylogenetic implications, may provide useful information for more practical aspects. Common buckwheat (*F. esculentum*) is a widely cultivated multipurpose crop [12]. Access to the chloroplast genome sequence may highlight other physiologically important traits in buckwheat. In addition, the chloroplast genome sequence can be utilized for developing species specific transformation vectors (for review see [13,14]). Therefore, the knowledge of the nucleotide sequence of buckwheat chloroplast genome opens up an avenue for the application of plastid genetic engineering to this plant.

## Methods

### Plant material

The seeds of *Fagopyrum *species used in this study were obtained from All-Russia Research Institute of Legumes and Groat Crops. Plant material for *Persicaria*, *Rheum*, *Reynoutria *and *Coccoloba *was obtained from Moscow State University Botanical garden.

### DNA extraction, amplification and sequencing

Total cellular DNA was isolated from fresh leaf tissue using NucleoSpin Plant DNA kit (Macherey-Nagel) following manufacturer's instructions. PCR amplification was performed on PTC-220 DNA Engine Dyad (MJ Research) using Encyclo PCR kit (Evrogen JSC, Moscow, Russia). For amplification, PCR conditions recommended in [15], i. e. 35 cycles of touchdown PCR with the decrease of annealing temperature from 55 to 50 deg C, were used. For some primer pairs, PCR was performed with optimization of gradient PCR involving altered annealing temperatures. For Long PCR, extension was performed at 68 deg C for 5 – 7 minutes, depending on the expected amplicon size. PCR products were purified using Gel Extraction & PCR Cleanup Kit (Cytokine Ltd, S.Petersburg, Russia). Automated sequencing was performed on ABI 3100 sequencer using the Big Dye Terminator v.3.1 sequencing kit (ABI, USA).

#### Sequencing strategy and primer design

The sequence of buckwheat chloroplast genome was obtained using a PCR based approach, similar to the ASAP method described earlier in [15]. The inverted repeat region of the *Fagopyrum *chloroplast genome was amplified and sequenced with the ASAP primers; the large and small single copy regions were amplified using PCR primers developed from the multiple alignment of known chloroplast genome sequences of angiosperms (primers are listed in Additional file 1). These universal primers enabled amplification of the entire chloroplast genome of *Fagopyrum *with overlapping PCR fragments ranging in size from 0.5 to 9 kb. Long fragments had to be generated due to a lack of sequence conservation in the aligned chloroplast genomes and few structural changes (for example, in the IR-LSC and IR-SSC junctions). The larger fragments were amplified and sequenced with taxon-specific primers using a primer walking approach (complete list of taxon-specific primers is available in Additional file 2).

### Contig assembly and annotation

Sequences generated from a primer pair were first aligned using Blast 2 sequences (bl2seq) tool [16], available at NCBI website to develop contigs which were then assembled using the BioEdit software [17]. Draft genome annotation was generated using the organelle annotation package DOGMA [18]. The predicted annotations were further verified using BLAST similarity search [19].

### Phylogenetic analysis

For the phylogenetic analysis, a set of 61 protein-coding genes derived from 44 chloroplast genomes was collected (Table 1). The species included in the analysis represent all major lineages of angiosperms for which the chloroplast genome sequences have been reported till date.

**Table 1:** Taxa included in phylogenetic analysis with GenBank accession numbers and references.

Taxon	GenBank accession number	Reference
**Gymnosperms **(outgroup)		
*Ginkgo biloba*	DQ069337–DQ069702	Leebens-Mack et al., 2005 [54]
*Pinus thunbergii*	NC_001631	Wakasugi et al., 1994 [66]

**Basal Angiosperms**		
*Amborella trichopoda*	NC_005086	Goremykin et al., 2003a [67]
*Chloranthus spicatus*	NC_009598	Hansen et al., 2007 [9]
*Illicium oligandrum*	NC_009600	Hansen et al., 2007 [9]
*Nuphar advena*	NC_008788	Raubeson et al. 2007 [27]
*Nymphaea alba*	NC_006050	Goremykin et al., 2004 [68]

**Magnoliids**		
*Calycanthus floridus*	NC_004993	Goremykin et al., 2003b [69]
*Drimys granadensis*	NC_008456	Cai et al., 2006 [47]
*Liriodendron tulipifera*	NC_008326	Cai et al., 2006 [47]
*Piper cenocladum*	NC_008457	Cai et al., 2006 [47]

**Monocots**		
*Acorus calamus*	NC_007407	Goremykin et al., 2005 [70]
*Dioscorea elephantipes*	NC_009601	Hansen et al., 2007 [9]
*Lemna minor*	DQ400350	Mardanov et al. 2008 [39]
*Oryza sativa*	NC_001320	Hiratsuka et al., 1989 [63]
*Phalaenopsis aphrodite*	NC_007499	Chang et al., 2006 [71]
*Typha latifolia*	DQ069337–DQ069702	Leebens-Mack et al., 2005 [54]
*Yucca schidigera*	DQ069337–DQ069702	Leebens-Mack et al., 2005 [54]
*Zea mays*	NC_001666	Maier et al., 1995 [65]

**Eudicots**		
*Arabidopsis thaliana*	NC_000932	Sato et al., 1999 [72]
*Atropa belladonna*	NC_004561	Schmitz-Linneweber et al., 2002 [73]
*Buxus microphylla*	NC_009599	Hansen et al., 2007 [9]
*Citrus sinensis*	NC_008334	Bausher et al., 2006 [56]
*Coffea Arabica*	NC_008535	Nalapalli et al., 2007 [74]
*Cucumis sativus*	DQ119058	Kim et al., 2006 [75]
*Daucus carota*	NC_008325	Ruhlman et al., 2006 [57]
*Eucaliptus globulus*	NC_008115	Steane, 2005 [76]
*Fagopyrum esculentum*	EU254477	this study
*Glycine max*	NC_007942	Saski et al., 2005 [77]
*Gossypium hirsutum*	NC_007944	Lee et al., 2006 [78]
*Helianthus annuus*	NC_007977	Timme et al., 2007 [79]
*Jasminum nudiflorum*	NC_008407	Lee et al., 2007 [40]
*Lactuca sativa*	NC_007578	Kanamoto et al., unpublished
*Lotus japonicus*	NC_002694	Kato et al., 2000 [80]
*Morus indica*	NC_008359	Ravi et al., 2006 [53]
*Nandina domestica*	NC_008336	Moore et al., 2006 [81]
*Nicotiana tabacum*	NC_001879	Shinozaki et al., 1986 [82]
*Oenothera elata*	NC_002693	Hupfer et al., 2000 [83]
*Panax ginseng*	NC_006290	Kim, Lee, 2004 [84]
*Platanus occidentalis*	NC_008335	Moore et al., 2006 [81]
*Populus alba*	NC_008235	Okumura et al., 2006 [85]
*Ranunculus macranthus*	NC_008796	Raubeson et al., 2007 [27]
*Spinacia oleracea*	NC_002202	Schmitz-Linneweber et al., 2001 [32]
*Vitis vinifera*	NC_007957	Jansen et al., 2006 [55]

Gene sequences were parsed to detect frameshift mutations and edited when necessary. Sequences were translated into derived amino acid sequences, which were further aligned using MUSCLE ver. 3.6 [20] followed by manual correction. Nucleotide sequence alignment was overlaid on the amino acid sequence alignment.

Phylogenetic analyses using maximum parsimony (MP) method was performed using PAUP* ver. 4.0b8 [21]. Bayesian inference of phylogeny was explored using the MrBayes program ver. 3.1.2 [22]. Alternative topologies test was performed with the Tree-Puzzle program [23].

MP analysis involved a heuristic search using tree bisection and reconnection (TBR) branch swapping and 100 random addition replicates. Non-parametric bootstrap analysis [24] was performed with 100 replicates with TBR branch swapping. Both nucleotide and amino acid sequences were included in the analysis.

Bayesian analysis was also performed using both amino acid and nucleotide sequence datasets. For nucleotide sequence analyses different partitioning strategies were employed: each gene as a separate partition (61 partitions), combination of genes according to their function (4 partitions: photosynthetic metabolism, photosynthetic apparatus, gene expression and others) and partitioning according to codon position (3 partitions). For each of the 61 amino acid partitions, the most appropriate model of substitutions was determined by the BIC (Bayesian Information Criterion) in Modelgenerator ver. 0.43 [25]. Similarly for each nucleotide partition, the most appropriate model of nucleotide substitution was determined by the AIC (Akaike Information Criterion) in Modeltest ver. 3.7 [26].

Bayesian analysis was performed with three chains in each of the two runs. Each chain started with a random tree, 2500000 replicates for amino acid data and 5000000 replicates for nucleotide data were generated. The trees thus obtained were sampled every 100 generations. The proportion of invariable sites and the shape of gamma-distribution rates were unlinked across partitions. The number of discarded trees was determined using convergence diagnostics.

## Results and Discussion

### Overall structure and gene content of buckwheat chloroplast genome

The GenBank accession number for the nucleotide sequence reported in this study is EU254477. Complete chloroplast genome of *Fagopyrum esculentum *ssp. *ancestrale *is composed of 159599 nucleotide bases. This exceeds the average size of flowering plants chloroplast genomes ~155 Kb and, in particular, almost 9 Kb larger then the chloroplast genome of its closest relative *Spinacia oleracea *(150725 bp). The observed increase in size is due to the expansion of the inverted repeat (IR) region. The size of the IR region is 30684, the large single copy (LSC) and the small single copy (SSC) regions are 84888 and 13343 bp respectively. Overall AT-content of the entire plastome is 62%, the LSC and SSC are 63% and 68% AT respectively and inverted repeat is 59% AT rich. This is comparable with the other land plant chloroplast genomes (*Spinacia oleracea *– 63%, *Nicotiana sylvestris *– 62%, *Lotus japonicus *– 64%, *Zea mays *– 62%). The lower AT% of the IR region reflects the lower AT-content of ribosomal RNA genes.

**Figure 1:**
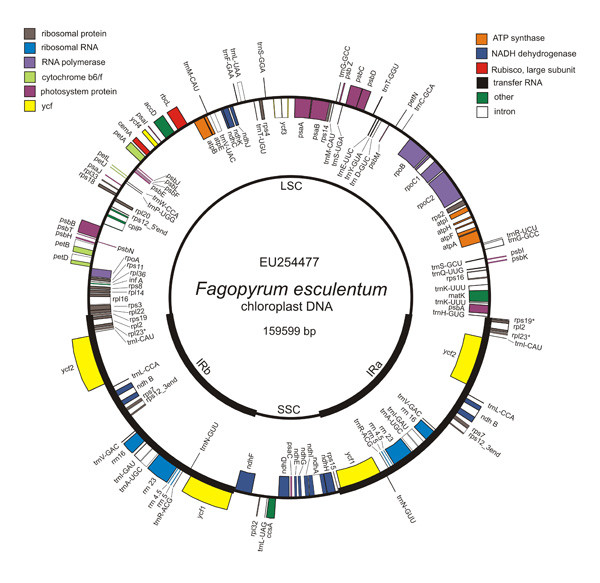
**Gene map of the *Fagopyrum esculentum *chloroplast genome**. The thick lines indicate the extent of the inverted repeats (IRa and IRb), which separate the genome into small (SSC) and large (LSC) single copy regions. Genes shown inside the circle are transcribed clockwise, those outside the circle are transcribed counterclockwise. Asterisk (*) indicates pseudogenes.

The gene order and content of the buckwheat chloroplast genome is identical to that of *Spinacia *(Fig. 1). This similarity is discernible not only in the functional genes but also in the pseudogenes. In both buckwheat and *Spinacia *chloroplast genome, the sequences representing *rpl23 *and *ycf15 *genes are interrupted by the internal stop codons, indicating their pseudogene status. The latter situation is quite commonly observed amongst angiosperms: detailed studies of the evolutionary pattern of this region have revealed that *ycf15 *gene is not a protein-coding gene [27]. On the contrary, *rpl23 *gene is known to be present and functional in most flowering plants [28]. Therefore, pseudogenization of this gene may represent a feature that is unique to caryophyllids. Thus far, four sequences of the *rpl23 *region have been reported for caryophyllids; beet (*Beta vulgaris*), spinach, buckwheat and *Silene latifolia*, (Caryophyllaceae). Comparative analysis of the four sequences reveals that all of them harbor mutations; however their exact structure is different (Additional file 3). In buckwheat there is a 4 bp insertion, which affects the reading frame, leading to the generation of a stop codon. *Beta *and *Spinacia *share a common 14 bp deletion, which also alters the reading frame. Interestingly in *Silene *this region seems to be less affected. It does not harbor any frameshift mutations however the gene has a stop codon near the 5'-end. This observation cannot serve as the sole evidence for this gene being non-functional as a stop codon can be edited to a sense codon by the commonly observed phenomenon of RNA editing in the chloroplasts. A sequencing artifact could be the other plausible explanation for the presence of the stop codon. To evaluate if the pseudogenization of *rpl23 *gene is a common structural feature of caryophyllid plastomes, additional sequence information from other species is required. Transcription of the *rpl23 *gene has been experimentally demonstrated in spinach however protein products were not detected in this study [29]. Therefore it is plausibly an expressed pseudogene. A similar situation may exist in the case of buckwheat as well.

Another feature that is shared between *Fagopyrum *and *Spinacia *plastomes is the presence of two genes – *psbL *and *ndhD *– with an ACG initiation codon. C to U RNA editing-mediated creation of the AUG initiation codon from the original ACG codon is a common phenomenon observed in case of chloroplast genome-derived transcripts. RNA editing has been experimentally demonstrated for *psbL *[30] and *ndhD *genes in tobacco. The editing of *ndhD *gene has also been reported in spinach and snapdragon [31]. Since *psbL *and *ndhD *genes code for proteins essential for chloroplast function, it is safe to assume that their transcripts are edited in *Fagopyrum*. This can be substantiated with the help of cDNA sequencing or other experimental evidence in future studies. Interestingly, there are two structural features that are unique to the *Fagopyrum *plastome. First is the position of the IR-SSC borders relative to other plastid genomes. In *Spinacia *the IRa/SSC border resides with in the 3' region of *ycf1 *gene. The remaining *ycf1 *gene lies in the SSC; a copy of the 3'region of *ycf1 *gene located in the IRb produces the *ycf1 *pseudogene at the IRb/SSC border. Buckwheat possesses an expanded IR region and two full-length copies of *ycf1 *gene are thus generated.

Second, buckwheat plastid genome is comprised of 18 intron-containing genes compared to spinach in which the number of these genes is reduced to 17. This difference is due to the loss of *rpl2 *intron in spinach [32]. The loss of intron in *rpl2 *gene has occurred independently in several lineages of flowering plants [33] and it is considered to be a characteristic feature observed in the members of the core Caryophyllales. Thus, the presence of the *rpl2 *intron in buckwheat (Polygonaceae) emphasizes the distinction of this group from the core Caryophyllales. The *rpl2 *intron has also been reported in *Rumex*, another representative of Polygonaceae, and in the members of Plumbaginaceae, a family that is close to Polygonaceae and often associated with Caryophyllales [33].

### Expansion of the inverted repeat region in buckwheat chloroplast genome

The size and thus the boundary of the chloroplast genome inverted repeat (IR) region is variable amongst different plant species [34]. Previous studies on IR borders have mainly focused on the IR and the LSC junction [34,35] that revealed multiple instances of expansion and contraction in the IR region, ranging from a few base pairs to more than 15 Kb.

The expanded IR region in buckwheat represents an average increase of 5 Kb when compared with other flowering plants. The observed expansion and the sequence of the enhanced IR region in other *Fagopyrum *species has been reported previously [36,37]. This region was shown to be highly similar to the small single copy (SSC) region adjacent to IRa in other dicot chloroplast genomes which enabled the conclusion that this expansion is due to the inclusion of the SSC sequences.

**Figure 2:**
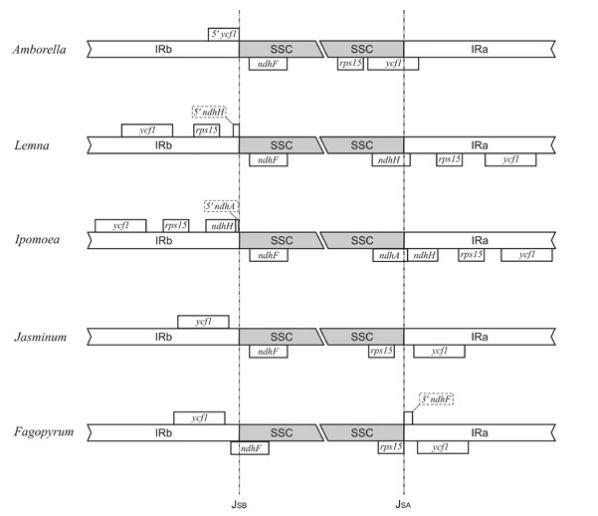
**Structure of IRb/SSC and SSC/IRa in Amborella and in angiosperms with expanded IR**. In *Amborella *IR/SSC junction occurs within *ycf1*; truncated copy of *ycf1 *is thus generated at the IRb/SSC border. Such organisation of IR/SSC junction is characteristic for most angiosperms. *Lemna*, *Ipomoea*, *Jasminum *and *Fagopyrum *represent different ways of the IR expansion. *Jasminum *and *Fagopyrum *both have included *ycf1 *in the IR. In *Lemna *not only *ycf1*, but also *rps15 *and a part of *ndhH *belong to the IR, and in *Ipomoea *overall *ndhH *and a part of *ndhA *are also duplicated.

The sequence information generated in the present study confirms the expansion of the IR in *Fagopyrum esculentum *ssp. *ancestrale*. In contrast to most angiosperms, the expansion encompasses the *ycf1 *gene a conserved chloroplast ORF found in all known dicot and some monocot chloroplast genomes. Its exact function is unknown but together with another conserved chloroplast ORF *ycf2*, it has been shown to be vital for chloroplast function in tobacco [38]. In most instances, the IR region contains only a part of the *ycf1 *gene with the other part located in the SSC region of the plastome. The length of *ycf1 *gene that is duplicated ranges from 156 bp in *Nymphaea *to 1583 bp in *Amborella *[27]. However, inclusion of the *ycf1 *gene in the IR region was also reported in a monocot *Lemna minor *[39], in an asterid *Jasminum nudiflorum *[40] and *Ipomoea purpurea *[41]. Given that these taxa belong to diverse lineages of flowering plants, the expansion of *ycf*1 gene has most probably appeared independently in each of these groups from an ancestral *Amborella*-like chloroplast genome. Moreover, the exact mode of *ycf*1 gene expansion varies in different species (Fig. 2). For example, in *Jasminum *the IR/SSC border is positioned within the *ycf1*-*ndhF *spacer region on one border and the other border is positioned within the *rps15*-*ycf1 *spacer. In *Lemna *the duplication encompasses *rps15 *gene and 5' region of the *ndhH *gene. In *Ipomoea *this expansion extends even further and includes the complete *ycf1 *gene, *rps15 *gene, *ndhH *gene, and a short region of *ndhA *gene's first exon. In case of buckwheat the expanded region includes the *ycf1*-*ndhF *spacer and 70 bp of the 3' end of *ndhF *gene.

It is interesting to note that the sequence of the expanded region in *Fagopyrum esculentum *ssp. *ancestrale *is different from the sequence reported by Aii et al. [37]. The authors had reported a 3711 bp inversion within this region. This inversion affected the transcriptional continuity of *ycf1 *gene, causing an interruption in its reading frame. The observed difference in the sequence reported in this study could be plausibly explained by the use of different genotypes of buckwheat. However, the reported inversion could be potentially employed for tracing the origin of various buckwheat cultivars. Importantly, the set of buckwheat-specific primers reported in this work are expected to enable future studies of this and other potentially interesting structural features.

We further investigated the expansion of the IR in other species related to *Fagopyrum esculentum *spp. *ancestrale *using a PCR based approach with two sets of primers (for details see Additional file 4). One primer in each set annealed to the *ndhF *and *rps15 *genes within the SSC. The other primer is common to both sets and anneals to the *ycf1 *gene. It has only one annealing site within the SSC in chloroplast genomes that do not possess the IR expansion (e. g., *Spinacia*), but two annealing sites (in the direct and reverse orientations) in the IR of the species which have two copies of *ycf1 *due to the IR expansion (like *Fagopyrum*).

**Figure 3:**
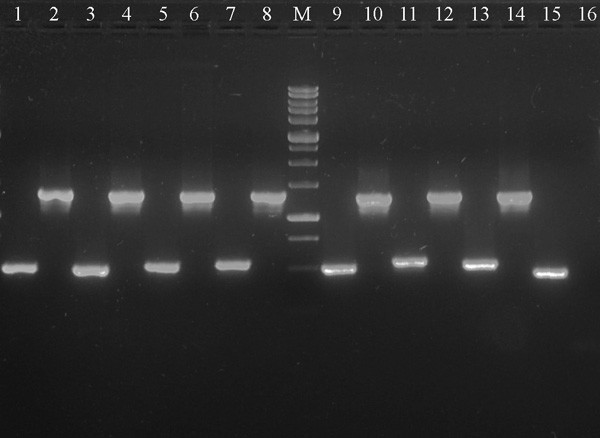
**Expansion of the IR region in Polygonaceae**. Ethidium bromide stained 1.5% agarose gel showing PCR amplification of *rps15*-*ycf1 *and *ycf1-ndhF *spacers for selected Polygonaceae taxa compared to *Spinacia oleracea*. 1, 2 – *Fagopyrum esculentum *ssp. *ancestrale rps15*-*ycf1 *and *ycf1*-*ndhF *fragments respectively, 3, 4 – *F. homotropicum *SSC *rps15*-*ycf1 *and *ycf1*-*ndhF*, 5, 6 – *F. tataricum rps15*-*ycf1 *and *ycf1*-*ndhF*, 7, 8 – *Persicaria macrophylla rps15*-*ycf1 *and *ycf1*-*ndhF*, 9, 10 – *Rheum tanguticum rps15*-*ycf1 *and *ycf1*-*ndhF*, 11, 12 – *Reynoutria japonica rps15*-*ycf1 *and *ycf1*-*ndhF*, 13, 14 – *Coccoloba uvifera rps15*-*ycf1 *and *ycf1*-*ndhF*, 15, 16 – *Spinacia oleracea rps15*-*ycf1 *and *ycf1*-*ndhF *(*ycf1*-*ndhF *– no amplification). M is the 0.25 – 10 Kb DNA ladder (SibEnzyme Ltd, Moscow, Russia), lowermost visible lane corresponds to 0.5 Kb.

These studies revealed that the observed expansion of the IR was present in all Polygonaceae members sampled in the study that included two additional buckwheat species examined besides *F. esculentum *(*F. tataricum *and *F. homotropicum*). Similar IR expansion was observed in *Persicaria*, *Rheum, Reinoutria *and *Coccoloba *species as well (Fig. 3). For buckwheat species the expansion of the IR was further confirmed by sequencing of the IRb/SSC and SSC/IRa borders (accession numbers EU272335 – EU272336 for *F*. *tataricum *and EU272337 – EU272338 for *F. homotropicum*). Thus from these studies it is clear that the expansion is not only a characteristic of *Fagopyrum*, but also for some other related genera and this may represent a common feature of Polygonaceae. Comparative analysis of the various sequences derived from buckwheat species revealed minor variations in the fine structure of the IR/SSC border. In *F. esculentum *and *F. homotropicum *SSC/IR borders are identical and lie within the *ndhF *gene (IRb/SSC) and 2 bp upstream of the *rps15 *gene (SSC/IRb). Overall the *rps15*-*ycf1 *spacer region is included in the IR in the above-mentioned species while in *F. tataricum rps15 *gene and 21 bp of *rps15*-*ycf1 *intergenic spacer region are located in the SSC. Based on the phylogenetic analysis of nuclear and chloroplast loci *F. esculentum *and *F. homotropicum *are closely related to each other [42,43]. The fine structure of IR/SSC borders in these species is consistent with these data and further studies of this region can be of utility to illustrate additional phylogenetic relationships within *Fagopyrum*.

### Phylogenetic analysis

In order to determine the relative position of *Fagopyrum *amongst angiosperms comparative phylogenetic analyses of available plastid genome sequences was performed. The data set consisted of 61 concatenated protein-coding gene sequences from 44 different taxa, including two gymnosperm species as outgroups. Nucleotide and amino acid data sets contained 42504 and 14168 characters respectively after the exclusion of ambiguous alignment positions.

**Figure 4:**
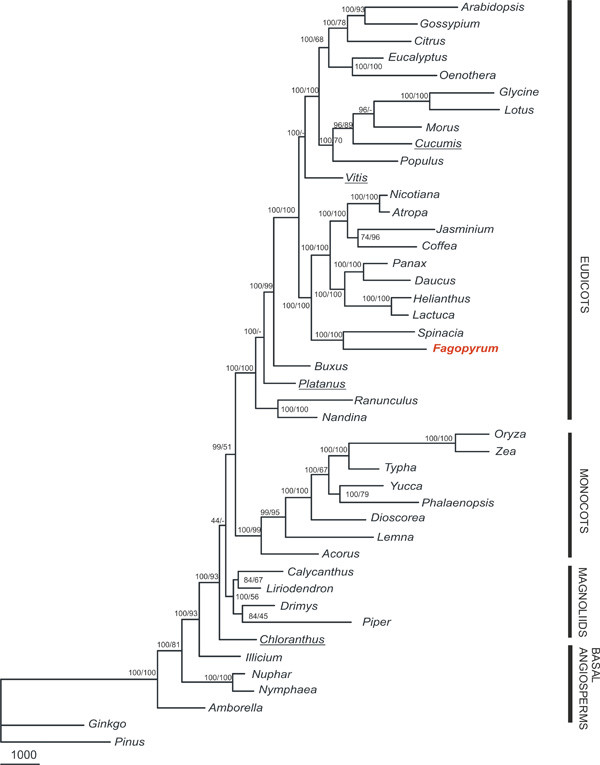
**Maximum parsimony phylogenetic tree**. This tree is based on nucleotide sequences of 61 protein-coding genes from 44 taxa. Tree length is 85896, consistency index is 0.41 and retention index is 0.48. Numbers at nodes indicate bootstrap support values; first number is for nucleotide sequence data set, second is for amino acid sequence data set. Species which differ in position according to the analysis of these two types of data are underlined. Branch lengths are proportional to the number of expected nucleotide substitutions; scale bar corresponds to 1000 changes.

Maximum parsimony (MP) analyses of all aligned nucleotide positions resulted in a single fully resolved tree, in which most of the nodes gained high support in bootstrap analyses (Fig. 4) except for the placement of *Chloranthus*. MP analysis of amino acid data also produced a single tree, but its topology was different for the placement of *Vitis *(which became a sister to rosids and asterids with boothstrap support of 100%), *Cucumis *(it forms a cluster with *Morus *with a bootstrap support of 64%), *Platanus *(sister to Ranunculales with low bootstrap support) and *Chloranthus *(sister to magnoliids with low bootstrap support).

**Figure 5:**
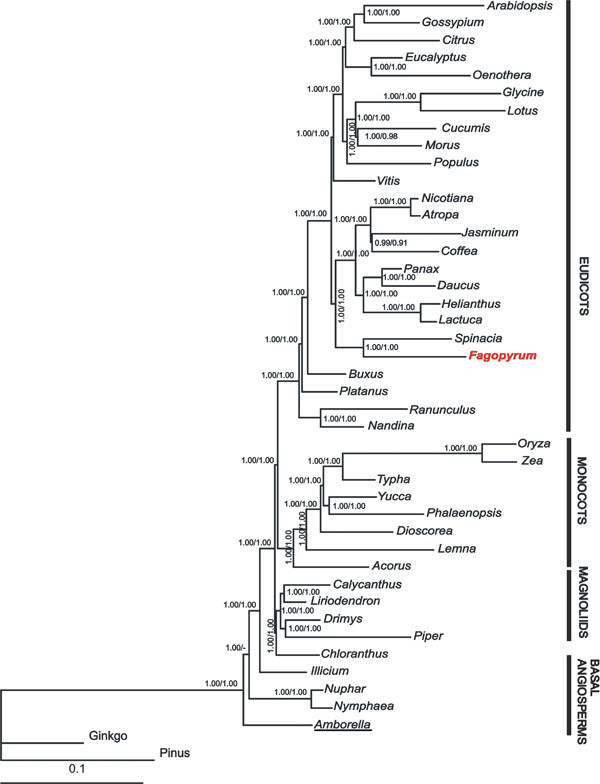
**Bayesian tree**. This tree is inferred from the analysis of nucleotide data set, all codon positions are included, and each gene represents separate partition. Numbers at nodes indicate posterior probability, first number is for posterior probabilities inferred from the analysis of all codon position, second is for posterior probabilities inferred from the analysis of first two codon positions. Branch lengths are proportional to the number of expected nucleotide substitutions; scale bar corresponds to one substitution per ten sites. Species which differ in position according to the analysis of all and first two codon positions are underlined.

The topologies of the Bayesian trees derived from the partitioned nucleotide matrix and amino acid sequence analyses were identical (Fig. 5) regardless of the chosen partitioning scheme. Exclusion of the third codon position from the Bayesian analysis resulted in a similar tree with the exception of *Amborella *uniting with Nymphaeales.

It has been demonstrated previously that the partitioning of complex datasets greatly improves the performance of Bayesian inference [44-46]. Thus, we employed different partitioning schemes (61 partitions, 4 partitions and 3 partitions) in our analyses however, comparison of the resulting trees showed no difference in the resulting tree topologies. At the same time dividing the dataset into 3 partitions seemed more logical if harmonic means of marginal likelihoods were compared (-455638.67, -455391.40, -449073.19 for 61, 4 and 3 partitions, respectively).

In general, our inferred chloroplast phylogenies are very similar to recently published molecular trees, in which the interrelationships and the monophyly of magnoliids, monocots and eudicots were strongly supported [9,10,47,48]. In all the derived phylogenetic trees, *Fagopyrum *and *Spinacia *(both represent Caryophyllales *sensu lato*) form a sister clade to asterids. To determine if alternative topologies with placement of Caryophyllales as a sister group to rosids (AT1) or to rosids and asterids (AT2) can be distinguished, a Shimodaira-Hasegawa test [49] was conducted and expected-likelihood weights [50] were calculated using RELL optimization [51]. According to the results of these tests, the alternative placement of Caryophyllales is significantly worse than the optimal topology, supporting a close relationship of Caryophyllales and asterids (p < 0.001, c<0.001 for both AT1 and AT2).

A clade of rosids, with grape being basal among them, is sister to asterids and Caryophyllales. Notably in our dataset inclusion of *Morus *sequence leads to stabilization of *Cucumis *position and in all our analyses the eurosids I are monophyletic. In the most recently published phylogenetic trees based on chloroplast sequences, the placement of *Cucumis *was unstable. It either united with Myrtaceae (*Eucalyptus, Oenothera*) in the bayesian trees or nested within eurosids I in the MP trees [9,47,52]. A close relationship of *Morus *and *Cucumis *has been demonstrated previously [53] and in our Bayesian trees these species form a cluster with a high posterior probability value. Such grouping is questionable based on our MP analysis and a previously reported analysis of 64 plastid genomes [10] that place them as a grade. However, it is obvious that eurosids I may be considered monophyletic.

*Ranunculus *and *Nandina *appear to constitute the basal most clade within eudicots. *Buxus *is the closest ally to core eudicots in all the derived trees, whereas the position of *Platanus *among basal eudicots cannot be firmly determined. Its intermediate position is not supported in the MP analysis with bootstrap resampling of amino acid sequences.

*Amborella*, Nymphaeales, Chloranthales and magnoliids remain the most problematic groups in angiosperm phylogeny. Their phylogenetic relationships are still equivocal and are dependent on the method and data used during analyses. None of the alternative hypotheses concerning *Amborella *(a sister to Nymphaeales or to all other angiosperms) and *Chloranthus *(a sister to magnoliids only or to magnoliids, monocots and eudicots) can be rejected on the basis of phylogenetic analysis of the chloroplast genome sequences [9,10]. Obviously, limited taxon sampling is the first weakness of the chloroplast phylogeny analyses at the current stage, therefore the problem cannot be resolved until additional representatives from basal angiosperms and gymnosperms are included in the analyses to obtain robust relationships. The same holds true for monocots and eudicots as long as many important lineages are missing. One should not be deluded by high bootstrap or posterior probability values, because high support for a doubtful clade is often obtained when a very large number of characters (like whole chloroplast genome sequences) but small number of taxa are used in phylogenetic analysis. For example, *Calycanthus *in some trees was placed as a sister to eudicots with high or moderate support [53-57] until additional magnoliids were included in the analyses [47]. To overcome the issue of taxon sampling, it is worth identifying genes that are most efficient for phylogenetic analysis and then to analyze these genes in more taxa. Recently *RPO *genes, coding for plastid RNA-polymerase subunits, have been shown to generate the topology of a phylogenetic tree similar to the whole plastid genomes phylogeny [58]. Another example is the use of slowly evolving genes encoded in the chloroplast inverted repeat region that have helped to resolve phylogenetic relationships within Saxifragales [1].

### Distribution of ACG initiation codon in rpl2, psbL and ndhD genes in angiosperms in the phylogenetic context

Several chloroplast genes in angiosperm plastomes are known to possess an atypical initiation codon ACG. For some species (tobacco, snapdragon, spinach, maize) there is strong experimental evidence that this codon is edited to the standard AUG codon [30,31,59] suggesting that similar mechanism may exist in other species. Since RNA editing patterns are thought to be the subjects of extensive parallel evolution, they are not necessarily phylogenetically informative [60]. However, for three plastid genes – *rpl2*, *psbL *and *ndhD *– a correlation between RNA editing and phylogeny has been reported [61]. This study was based on sequences from 7 angiosperms plastomes; it was concluded that RNA editing in *psbL *gene emerged in a common ancestor of angiosperms that was then lost in monocots. Editing of *rpl2 *gene emerged only in monocots and for *ndhD *gene it was observed only in dicots [61].

The availability of a large number of complete chloroplast genome sequences from angiosperms and improved knowledge about flowering plant phylogeny allows re-evaluation of this conclusion. We performed a survey of the potential RNA editing sites in the initiation codons of *rpl2*, *psbL *and *ndhD *in 44 seed plant chloroplast genomes sequenced till date (including *Fagopyrum*) and studied its distribution in different evolutionary lineages using phylogenetic trees reported in this article as a framework (Additional file 5). Foremost, our analyses indicate that among seed plants the ACG initiation codon in *rpl2 *gene is not only a characteristic of monocots, but also of some early divergent angiosperms (*Amborella*, *Chloranthus *and magnoliids) and lower eudicots (Ranunculales and *Platanus*). However, it is absent in *Illicium *and Nymphaeales that are also the representatives of basal lineages of angiosperms. Thus, it is difficult to conclude that the RNA editing at this site emerged in a common ancestor of angiosperms and then was lost in *Illicium *and Nymphaeales. Alternatively, it may have evolved later, after the divergence of the members of ANITA grade and its occurrence in *Amborella *may be due to parallel evolution. The editing in *ndhD *gene seemed to have evolved in a common ancestor of angiosperms and then lost in *Nymphaea *and in some monocots. In all the grasses studied till date *ndhD *gene has a standard ATG initiation codon [62-65] suggesting that it may be common for all the members of this family. The pattern of distribution of the ACG initiation codon in *psbL *gene is more complex. Its presence is characteristic for most of the early divergent angiosperms (except for *Chloranthus *and *Calycanthus*), but absent in all sampled monocots and in several different lineages of eudicots. We assume that the presence of the ACG initiation codon (and, presumably, RNA editing) in this gene is an ancestral character state for angiosperms and its absence is due to multiple parallel losses. However this observation needs to be verified further using broader taxon sampling.

## Conclusion

Complete sequence of buckwheat (*Fagopyrum esculentum *ssp. *ancestrale*) chloroplast DNA has been generated using a PCR based approach validating the utility of this approach especially for non-rearranged angiosperm chloroplast genomes. This represents the first sequenced plastid genome of the family Polygonaceae and of non-core Caryophyllales. Gene content and order of buckwheat plastome is similar to that of *Spinacia oleracea*, its relative from core Caryophyllales. However two structural differences have been revealed. First of them is the presence of an intron in *rpl2 *gene and the second is the expansion of inverted repeat region. The exact structure of the expanded region is different from previously reported data and includes an intact ORF for the *ycf1 *gene.

Phylogenetic analysis of 61 protein-coding genes in 44 taxa, including newly obtained chloroplast genome sequence (*Fagopyrum*) provides strong support for the sister relationships of Caryophyllales sensu lato (including Polygonaceae) and asterids. Most of other conclusions from previous phylogenetic studies of chloroplast genomes are also confirmed, in particular the placement of *Amborella *(or *Amborella *+ Nymphaeales) as a basalmost angiosperm, sister relationships of *Chloranthus *and magnoliids and the position of Ranunculales (*Ranunculus *+ *Nandina*) as the earliest diverging lineage of eudicots.

The study of distribution of the potential RNA editing sites in the initiation codon of *rpl2*, *psbL *and *ndhD *genes in angiosperms reveals some correlations with the phylogeny though confirms that the evolution of RNA editing is a subject of extensive parallel changes.

## Abbreviations

APG: Angiosperm phylogeny group; ASAP: Amplification, sequencing, annotation of plastomes; bp: base pair; cp: chloroplast; DOGMA: Dual organellar genome annotator; IR: inverted repeat; Kb: kilobase, LSC: large single copy, MP: maximum parsimony; ORF: Open reading frame; PCR: Polymerase chain reaction; SSC: small single copy; TBR: tree bisection-reconnection.

## Authors' contributions

MDL participated in the design of the study, designed universal and taxon-specific primers for amplification and sequencing, performed all PCR reactions, participated in contig assembly and performed the annotation and prepared the initial draft. THS performed phylogenetic analysis and wrote phylogenetic part of the manuscript. AD provided the ASAP primers for the amplification of the inverted repeat region, edited and contributed to the writing of the manuscript. AAP participated in the design of the study, contig assembly, and developed the figures. All authors read and approved the final manuscript.

## Supplementary Material

Additional file 1 Conserved primers developed for amplification and sequencing of buckwheat chloroplast genome. Table. Click here for file

Additional file 2 Taxon-specific primers. Contains the list of buckwheat-specific primers. Primers are named according their position in buckwheat chloroplast genome. For example, for the primers 4080F and 4621R 4080 and 4621 are the starts of the primer sequences on forward and reverse strands, respectively. Primers annealing at the IR region have double name according their position on both IRa and IRb. Click here for file

Additional file 3 Alignment of *rpl23 *homologs in angiosperms and gymnosperms, illustrating the mutations in *rpl23 *in Caryophyllales. *Beta *and *Spinacia *have 14 bp deletion (alignment positions 131–145), *Silene *has a substitution in 17 alignment position. This substitution (G instead of T or C) creates a stop codon TAG. *Fagopyrum *has and insertion of 4 bp (alignment positions 49–53). Click here for file

Additional file 4 Details of the PCR assay of IR expansion, including primer locations and expected amplicon lengths. Table. Click here for file

Additional file 5 Distribution of potential RNA editing site in *rpl2*, *psbL *and *ndhD *in angiosperms. Filled squares denote the presence of ACG initiation codon, thin squares – the presence of typical ATG initiation codon. Blue color is for *rpl2*, red for *psbL *and black for *ndhD*. Question marks denote ambiguous character state (due to the loss of gene or the lack of sequence data). Phylogenetic tree is inferred from maximum parsimony analysis of nucleotide data set. Click here for file
