# Supplementary material for: Comparative chloroplast genomics and phylogenetics of Fagopyrum esculentum ssp. ancestrale – A wild ancestor of cultivated buckwheat
Source: BMC Plant Biol. 2008 May 20;8:59. doi: 10.1186/1471-2229-8-59 (PMC2430205; doi:10.1186/1471-2229-8-59)
Supplement: Additional file 1 — Conserved primers developed for amplification and sequencing of buckwheat chloroplast genome. Table. [file 1471-2229-8-59-S1.doc]

| primer name | sequence (5’-3’) | position in *Spinacia* cp genome | | position in *Fagopyrum* cp genome | genomic region |
| --- | --- | --- | --- | --- | --- |
| **large single copy region** | | | | | |
| LSC-1F | CGCGCATGGTGGATTCACAATCC | 21 –  45 | | 25 –  47 | trnH |
| LSC-1R | GTTATGCATGAACGTAATGCTC | 270 –  291 | | 490 –  511 | psbA |
| LSC-1R1 | GATGGTTTGGTGTTTTGATGATCCCTA | 1161 – 1187 | | 1381 – 1407 | psbA |
| LSC-2F | AACAGGTTCACGAATACCATCAATA | 1078 – 1102 | | 1298 – 1322 | psbA |
| LSC-2R | CAAGGAAAATCAATTCTGGCTT | 2442 – 2463 | | 2636 – 2657 | matK |
| LSC-3F | ACATTTGACTCCGTATTACTGAM | 2256 – 2278 | | 2450 – 2472 | matK |
| LSC-3R | CTTGGTTCAAACTCTTCGCTACTG | 2801 – 2824 | | 2992 – 3015 | matK |
| LSC-4F | TATAGGAACAATAAGAATCTTTGATTC | 2666 – 2692 | | 2860 – 2886 | matK |
| LSC-4R | GGGTTGCTAACTCAACGGTAGA | 4034 – 4055 | | 4242 – 4263 | trnK, 2 exon |
| LSC-5F | GTAAAAGATTCTAGCCGCACTT | 3999 – 4020 | | 4207 – 4228 | trnK, 2 exon/1 intron |
| LSC-5R | GAGCCGTCTATCGAATCGTTGCAATT | 4866 – 4891 | | 5214 – 5239 | rps16, 2 exon |
| LSC-6F | ATAGCAGGAACATTTAAATAAGTTTGA | 4774 – 4800 | | 5112 – 5146 | rps16, 2 exon |
| LSC-6R | ATGGTAAAACTTCGTTTGAAAC | 5785 – 5806 | | 6116 – 6137 | rps16, 1 exon |
| LSC-7F | GTTGCTTTCTACCACATCGTT | 5767 – 5787 | | 6098 – 6118 | rps16, 1 exon |
| LSC-7R | AAAACTTACAGCAGCTTGCCAAAC | 6755 – 6778 | | 6977 – 7000 | psbK |
| LSC-8F | ATGCTTAATATCTTTAGTTTGATC | 6605 – 6628 | | 6821 – 6844 | psbK |
| LSC-8R | ATTGCTAATCCGTTGTACGAGTTA | 7514 – 7537 | | 7559 – 7582 | trnS |
| LSC-9F | AGAGAGGGATTCGAACCCT | 7486 – 7504 | | 7531 – 7549 | trnS |
| LSC-9R | ACCTATACCAAAGGTTTAGAAGA | 9072 – 9094 | | 9492 – 9514 | trnR |
| LSC-10F | CAAGCTAACGATGCGGGTTCGATTC | 8898 – 8922 | | 9314 – 9338 | trnG, 2 exon |
| LSC-10R | CAAATGTCTCTTCTATTAMGAAGACC | 9863 – 9888 | | 10383 – 10498 | atpA |
| LSC-11F | GAAATTACATTAGTRGGAATATA | 9695 – 9717 | | 10215 – 10237 | atpA |
| LSC-11R | TAATACCGATATTTTAGCAACAAAT | 12001 – 12024 | | 12505 – 12529 | atpF, 1 exon |
| LSC-12F | TTTTCGATTATCTAATAAATCAYTTA | 11154 – 11179 | | 11681 – 11706 | atpF, 2 exon |
| LSC-12R | GGCTTGCTTCTATTGGACCT | 12659 – 12678 | | 13175 – 13194 | atpH |
| LSC-13F | ATAAATWGTTAAMGCTTCCATAAA | 12533 – 12556 | | 13049 – 13073 | atpH |
| LSC-13F1 | GAGCCTGAATACCACTTGTAAATAATCC | 13482 – 13509 | | 13966 – 13933 | atpI |
| LSC-13R | GAATTAGTAGTTGTTGTTCTTGTTTCT | 13549 – 13575 | | 14033 – 14059 | atpI |
| LSC-16F | GCTTCGGATATAAAACTNTGAGTATT | 15615 – 15641 | | 16150 – 16175 | rpoC2 |
| LSC-17F | GAATCATACCATTTGTCYTTACTTCAAC | 16868 – 16895 | | 17390 – 17417 | rpoC2 |
| LSC-20F | TATTTCTTCGTTTYTTTGCCAATAAAT | 17735 – 17761 | | 18245 – 18271 | rpoC2 |
| LSC-20R | TGGTATTCTACAAGTGAATATTTGCG | 19096 – 19121 | | 19630 – 19655 | rpoC2 |
| LSC-21F | CTTGTGGATCCGACATTAATCCTCTCA | 18959 – 18985 | | 19493 – 19519 | rpoC2 |
| LSC-21R | CTATTTGTTTACATCCATTAGTT | 20179 – 20201 | | 20735 – 20757 | rpoC1 |
| LSC-22F | AGATTCATATGAGAAAACATAAGTAA | 20069 – 20094 | | 20625 – 20650 | rpoC1 |
| LSC-22R | GAACAATTAGCCGATYTGGATTTG | 21001 – 21024 | | 21545 – 21568 | rpoC1 |
| LSC-23F | TTCTATATTTGTTCGAAKAAAATG | 20845 – 20868 | | 21401 – 21424 | rpoC1 |
| LSC-23R | CCTATAAAAAGTGGAATTTGTGC | 22172 – 22194 | | 22725 – 22747 | rpoC1 |
| LSC-24F | ACTAGGAAGACGTTTYAAATACCA | 22009 – 22032 | | 22562 – 22585 | rpoC1 |
| LSC-24R | GCTATACTTGGCATTCAGRTATC | 23316 – 23338 | | 23869 – 23891 | rpoB |
| LSC-25F | GGATCCATCGCACATCAATRAC | 23245 – 23266 | | 23798 – 23819 | rpoB |
| LSC-25R | CGATATGAATCAYTTGAAAMATAA | 24684 – 24706 | | 25237 – 25260 | rpoB |
| LSC-26F | CTGAGGAGTCGGTATYAATTTATG | 24560 – 24583 | | 25113 – 25136 | rpoB |
| LSC-26R | CGACACCCGGATTTGAACTGG | 26765 – 26785 | | 27418 – 27439 | trnC |
| LSC-27F | TTTGGCGGCATGGCCGAG | 26715 – 26732 | | 27368 – 27385 | trnC |
| LSC-27F1 | TGGGCTGCTTTAATGGTAGT | 27306 – 27325 | | 28331 – 28350 | petN |
| LSC-27R | AAGTAAATATTCTYCGCATTTATTGCTAC | 28339 – 28366 | | 29239 – 29266 | psbM |
| LSC-28F | AAAAAAGCRGTAGGAACTAGAAT | 28306 – 28328 | | 29206 – 29228 | psbM |
| LSC-28R | ATGCCCGAGCGGTTAATGGG | 29641 – 29660 | | 30829 – 30848 | trnY |
| LSC-29F | GAGCTGGATTTGAACCAGCG | 29589 – 29608 | | 30777 – 30796 | trnY |
| LSC-29R | GTATACCATGAAGTTACAAAGG | 31667 – 31688 | | 33041 – 33062 | psbD |
| LSC-30F | TATGGATGACTGGTTACG | 31560 – 31577 | | 32943 – 32951 | psbD |
| LSC-30R | GGAAGTAAAATYAATCCTTGTTC | 32762 – 32784 | | 34136 – 34158 | psbC |
| LSC-31F | AACGCTCTTTAATGGAACTTTA | 32557 – 32578 | | 33931 – 33952 | psbC |
| LSC-31F1 | AAGCTCAAGCATTTACTTTTCTRGT | 33447 – 33471 | | 34820 – 34845 | psbC |
| LSC-31R | ATAATTGACTGCATTGATCTCNGTAGC | 33746 – 33772 | | 35120 – 35146 | psbC |
| LSC-31R1 | GGTTCGAATCCCTCTCTCTC | 34190 – 34209 | | 35603 – 35622 | trnS |
| LSC-32F | GCTCCTTTAGGTTCTTTAAATTC | 33710 – 33732 | | 35084 – 35106 | psbC |
| LSC-32R | TAATCCAAGCCATAAWGATGTACC | 34770 – 34793 | | 36143 – 36166 | psbZ |
| LSC-33F | CAATTGGCTGTTTTTGCATTAATT | 34659 – 34682 | | 36032 – 36055 | psbZ |
| LSC-33R | TCGACGTTGYTTTTTGACCGGAAG | 35746 – 35769 | | 37061 – 37084 | rps14 |
| LSC-34F | GCTACCAAACTGCTCTACCCCGCG | 35470 – 35493 | | 36788 – 36811 | trnM |
| LSC-34R | TTGGTTCATCATGCTATTGC | 36704 – 36723 | | 38024 – 38043 | psaB |
| LSC-35F | AAGCCGAAATATCACAAGTACC | 36558 – 36579 | | 37878 – 37899 | psaB |
| LSC-35R | ATTTGGGATCCTCATTTTGGTCAACC | 37979 – 38004 | | 39299 – 39324 | psaB |
| LSC-36F | TTCCATTTCGGTTGTAGGTGTAACCA | 37790 – 37815 | | 39110 – 39135 | psaB |
| LSC-36R | TATATTCATAATGATACCATGAGTGC | 39171 – 39196 | | 40491 – 40516 | psaA |
| LSC-37F | CATGRATTGTAAATGCATGAAT | 38926 – 38947 | | 40246 – 40267 | psaA |
| LSC-37F1 | AATGGAACCAACCRGCAAAAAGCATYAA | 40021 – 40048 | | 41341 – 41368 | psaA |
| LSC-37R | TGGAACCTACATGCTGATGCTCA | 40392 – 40414 | | 41712 – 41734 | psaA |
| LSC-37R1 | ATGATTATTCGTTCGCCGGAACC | 40530 – 40552 | | 41850 – 41872 | psaA |
| LSC-38F | GCATCAGCATGTAGGTTCCAGATC | 40395 – 40418 | | 41715 – 41738 | psaA |
| LSC-38R | ATGAAACCGAGATAGTTACCT | 41593 – 41613 | | 43059 – 43079 | ycf3, 2 intron |
| LSC-38F1 | TCTCCCTGTCGAATGGCTTGTTCTC | 41272 – 41296 | | 42752 – 42776 | ycf3, 3 exon |
| LSC-38R1 | GAATTATTATGAAGCTATGCGAC | 42224 – 42246 | | 43694 – 43716 | ycf3, 1 intron |
| LSC-39F | GATCACTGCCATATTATTAAAAGCTTG | 42069 – 42095 | | 43539 – 43565 | ycf3, 2 exon |
| LSC-39R | CAATATCGTATTCGTTTAGAAGAAA | 44930 – 44954 | | 46350 – 46374 | rps4 |
| LSC-40F | GGATATGGCGAAATTGGTAGACGCT | 46214 – 46238 | | 48133 – 48157 | trnL, 1 exon |
| LSC-40F1 | AAAATCGTGAGGGTTCAAGTCCCTCTA | 46568 – 46594 | | 48687 – 48713 | trnL, 2 exon |
| LSC-40R | AACCAGATTTGAACTGGTGACACGA | 46978 – 47002 | | 49104 – 49128 | trnF |
| LSC-44R | TGTTCAGTACCGATTCTTATAGTAC | 48576 – 48600 | | 50717 – 50741 | ndhK |
| LSC-45F | TTTGTAAAGYCTCTATTCCTTGG | 48050 – 48072 | | 50212 – 50233 | ndhJ |
| LSC-45R | GGTTCGAGTCCGTATAGCCCTA | 50011 – 50032 | | 52336 – 52357 | trnV, 2 exon |
| LSC-46F | TAGGGCTATACGGACTCGAACCGTAGA | 50011 – 50037 | | 52336 – 52362 | trnV |
| LSC-46R | CCGTATGAAAGCAATACTCTAACCGCT | 50872 – 50898 | | 53170 – 53196 | trnM |
| LSC-46R1 | TCAGTTAGGTAGAGCACCTCGTTTACAC | 50640 – 50668 | | 52950 – 52976 | trnV |
| LSC-46R2 | GACGAGCTAGGACACGAGT | 51170 – 51188 | | 53396 – 53415 | atpE |
| LSC-47F | CTCAGCGGTTAGAGTATTGCTTTCATA | 50869 – 50895 | | 53167 – 53193 | trnM |
| LSC-47R | GGAAATGATCTTTACATGGAAATGA | 52391 – 52415 | | 54620 – 54644 | atpB |
| LSC-48F | CCAACTCTCATACGMGCTCC | 52291 – 52310 | | 54520 – 54539 | atpB |
| LSC-48R | CATAGTCAGGAGTATAATAAGTCAATTTG | 53884 – 53912 | | 56121 – 56149 | rbcL |
| LSC-49F | ATGTCACCACAAACAGAGACT | 53825 – 53845 | | 56062 – 56082 | rbcL |
| LSC-51R | CCTACTACAGATCCCATACTACC | 56997 – 57019 | | 58903 – 58925 | accD |
| LSC-52F | GTGAACAATGTGGATATCATTTGAAAAT | 56737 – 56764 | | 58643 – 58670 | accD |
| LSC-52R | TTTCCAGGRAATCCCCAACGAAAAAT | 58862 – 58887 | | 60876 – 60901 | ycf4 |
| LSC-52R1 | AGARATAAAGAAGCCATTGC | 58150 – 58169 | | 60076 – 60095 | psaI |
| LSC-53F | CCACAAGGGATCGTGATGTCTTTYTA | 58733 – 58758 | | 60747 – 60772 | ycf4 |
| LSC-53R | TTTATTGTGTCGCTTAAGTTATA | 60112 – 60134 | | 62341 – 62363 | cemA |
| LSC-54F | GGGTTACTAATTGGTGGAATRC | 59786 – 59807 | | 62015 – 62036 | cemA |
| LSC-54R | CCACCTACATATATCGGATATTTTA | 61185 – 61209 | | 63344 – 63368 | petA |
| LSC-55F | GATACTGTATTTGAAGCAGTTG | 60878 – 60899 | | 63037 – 63058 | petA |
| LSC-55R | TATTACTCATTTTTGTACTTGCTGTT | 62713 – 62738 | | 64751 – 64776 | psbL |
| LSC-55F1 | AAGGTTCAATTGTCCGAAATGAATT | 61583 – 61607 | | 63742 – 63766 | petA |
| LSC-55R1 | ATGGCCGATACTACTGGAAG | 62527 – 62546 | | 64562 – 64581 | psbJ |
| LSC-56F | CTTCCAGTAGTATCGGCCAT | 62527 – 62546 | | 64562 – 64581 | psbJ |
| LSC-56R | ATGTCTGGAAGCACAGGAGAACGTT | 63185 – 63209 | | 65223 – 65247 | psbE |
| LSC-57F | GACGAATAACCAACCCGCAATGAATA | 63096 – 63120 | | 65134 – 65159 | psbE |
| LSC-57R | ATTCCAAATAGAAAAACTTCAATCAT | 64645 – 64670 | | 66425 – 66450 | petG |
| LSC-58F | ACTCTAACTAGTTATTTYGGTTTT | 64389 – 64412 | | 66158 – 66181 | petL |
| LSC-58R | CGCATCTGGGAATAAACGATTRAT | 65740 – 65759 | | 67595 – 67618 | psaJ |
| LSC-59F | ATGCGAGATCTAAAAACATATCT | 65652 – 65674 | | 67511 – 67533 | psaJ |
| LSC-59R | GTAACAAAGATAAAATACGAGCTTG | 66827 – 66851 | | 68988 – 69012 | rps18 |
| LSC-60F | GGGATCGAATTGATTATAGAAACAT | 66699 – 66723 | | 68860 – 68884 | rps18 |
| LSC60F1 | CTATTTGTGCAAGTATTTTACGATT | 67265 – 67289 | | 69384 – 69408 | rpl20 |
| LSC-60R | ATGCCAACYATTAAACAACTTATTA | 68423 – 68447 | | 70538 – 70562 | rps12_5’end |
| LSC-61F | ATACACCCTAGTACATGTTCC | 69334 – 68354 | | 70449 – 70469 | rps12, 5’end |
| LSC-61R | TGTCTCCACATTGGATCAAGAAC | 71197 – 71219 | | 73541 – 73563 | psbB |
| LSC-61F1 | AGCGTGAGGGAATGCTAAACGTTTG | 69403 – 69427 | | 71574 – 71598 | clpP, 2 exon |
| LSC-61R1 | ATGCCTATTGGTGTTCCAAAAGT | 70582 – 70604 | | 72904 – 72926 | cplP, 1 exon |
| LSC-61F2 | GGGCTTCTGTTGCTGACATAAAA | 68621 – 68643 | | 70776 – 70798 | cplP, 3 exon |
| LSC-62F | TGCCTTGGTATCGTGTTCATAC | 71054 – 71075 | | 73398 – 73419 | psbB |
| LSC-62R | TCAATCCCAGCAAAAACATCTCT | 72472 – 72494 | | 74816 – 74838 | psbB |
| LSC-63F | CTTTGAAATCAGATGGTGTTTTTCG | 72353 – 72377 | | 74697 – 74721 | psbB |
| LSC-63R | GACGAATTATAAATTTCCAAAATAATAGA | 73306 – 73334 | | 75657 – 75685 | psbH |
| LSC-64F | AATTCGGAATATGGTAAAGTAGC | 73222 – 73244 | | 75573 – 75595 | psbH |
| LSC-65F | ATCGATGGTCRGCAAGTATGATGGT | 74529 – 74553 | | 76832 – 76856 | petB, 2 exon |
| LSC-65R | AGCCAATTTAGCTCTTAATACAGGATCRTT | 75903 – 75923 | | 78118 – 78217 | petD, 2 exon |
| LSC-66F | TGCTCGAGCCGGATGATGAAAAAT | 75801 – 75824 | | 78091 – 78114 | petD, 2 exon |
| LSC-66R | AGTTTAACTCCGAAAGAAGCACTT | 76817 – 76840 | | 79215 – 79238 | rpoA |
| LSC-67F | GGCAATTCTRATTGGTCAATAAAAAT | 76623 – 76651 | | 79021 – 79046 | rpoA |
| LSC-67R | GTTATTCATGTTCAAGCRAGTTT | 77843 – 77865 | | 80241 – 80263 | rps11 |
| LSC-68F | GCTCGTAATGCYGCATCTCTTCC | 77627 – 77649 | | 80025 – 80047 | rps11 |
| LSC-68R | GACCTGGTCTACGAATCTATT | 78816 – 78836 | | 81173 – 81193 | rps8 |
| LSC-69F | CCATATATAACATAAAATTTCTCCGCC | 78685 – 78711 | | 81042 – 81068 | rps8 |
| LSC-69R | GGTGGAAAAATATGGGTACGTAT | 79942 – 79964 | | 82285 – 82307 | rpl16 |
| LSC-70F | TGAATTGAGTTCGTATAGGCATTTT | 79751 – 79775 | | 82094 – 82118 | rpl16 |
| LSC-70R | ATGGGACAAAAAATAAATCCACT | 81888 – 81910 | | 84203 – 84225 | rps3 |
| LSC-71F | TAATTCGATAGCTTTTTTCATTGC | 81458 – 81481 | | 83773 – 83796 | rps3 |
| **small single copy region** | | | | | |
| SSC-1F | GCAAGAGCTAAAGTAGCTCCTAA | | 109067 – 109089 | 116820 – 116843 | ndhF |
| ndhF-SSC-IRb* | CTTCATGTATGGTTACCTGATGCTATGGA | | 109235 – 109263 | 116989 – 117017 | ndhF |
| SSC-1R | GTTGCAATTCTGGTTCTTATTTATAGTGA | | 109664 – 109692 | 117418 – 117446 | ndhF |
| SSC-2F | GTCGTGTAAACCAAAAACCTAT | | 109494 – 109515 | 117248 – 117269 | ndhF |
| SSC-2R | GAAGTRCGCTTTTTTGGAACTGCCAT | | 110959 – 110984 | 118697 – 118722 | rpl32 |
| SSC-3F | ATGGCAGTTCCAAAAAAGCGYACTTC | | 110959 – 110984 | 118697 – 118722 | rpl32 |
| SSC-3R | TGCCGCGACTCGGACTCGAA | | 112166 – 112185 | 119748 – 119767 | trnL |
| SSC-4F | GCCGCTATGGTGAAATTGGTAGA | | 112106 – 112128 | 119688 – 119710 | trnL |
| SSC-4R | GGAATGATATTAACTCCTATTTATTYATTATC | | 113690 – 113721 | 121258 – 121289 | ndhD |
| SSC-5F | GATAATRAATAAATAGGAGTTAATATCATTCC | | 113690 – 113721 | 121258 – 121289 | ndhD |
| SSC-5R | GTATTRGAAATGATACCTTGGGA | | 115280 – 115302 | 122854 –122876 | psaC |
| SSC-6F | CGRGTTGTTTCATGCCATAAATAAAC | | 115151 – 115176 | 122725 – 122750 | psaC |
| SSC-6R | ATGGATTTRCCTGGACCAATACATGAT | | 116668 – 116694 | 124229 – 124255 | ndhG |
| SSC-7F | GAATAAAGATATACACACAAGAACCA | | 116548 – 116573 | 124109 – 124134 | ndhG |
| SSC-7R | AAGAATTAGTAGCAGGTTATCAAAC | | 117953 – 117977 | 125586 – 125610 | ndhA, 2 exon |
| SSC-8F | AGAAATTTCCATCCAAGATTTAATA | | 117659 – 117683 | 125292 – 125316 | ndhA, 2 exon |
| SSC-8R | ACARAAGTACAAGCTATCAATTCTTTTT | | 119726 – 119753 | 127326 – 127353 | ndhA, 1 exon |
| SSC-9F | GCATATTCAGGTCCAATACGTTGTTGTAT | | 119569 – 119597 | 127169 – 127197 | ndhA, 1 exon |
| rps15-SSC-IRa* | TGTGCAATTCCAAATGTAAAGTAAGTCT | | 121229 – 121256 | 128804 – 128831 | rps15 |
| * These primers are developed for Long-PCR reactions and used in pair with IRb-25F, enabling amplification of the IRb-SSC and SSC-IRa junction regions. | | | | | |

Additional file 1.

Conserved primers developed for amplification and sequencing of buckwheat chloroplast genome.
