# Supplementary material for: Comparative chloroplast genomics and phylogenetics of Fagopyrum esculentum ssp. ancestrale – A wild ancestor of cultivated buckwheat
Source: BMC Plant Biol. 2008 May 20;8:59. doi: 10.1186/1471-2229-8-59 (PMC2430205; doi:10.1186/1471-2229-8-59)
Supplement: Additional file 2 — Taxon-specific primers. Contains the list of buckwheat-specific primers. Primers are named according their position in buckwheat chloroplast genome. For example, for the primers 4080F and 4621R 4080 and 4621 are the starts of the primer sequences on forward and reverse strands, respectively. Primers annealing at the IR region have double name according their position on both IRa and IRb. [file 1471-2229-8-59-S2.doc]

| primer name | sequence (5’-3’) |
| --- | --- |
| **large single copy region** | |
| 4080F | ACGACCTTTTTTCAAAATTCTCCGTTGA |
| 4621R | GTGCCAATCCAACACAAGTTCTTTC |
| 5401R | GATTTTGTGGATCTATTTGTCATTCGA |
| 6003F | CTAACAGAACAGGGGATGTCGAGT |
| 6208R | ACTAAACCAATGACTATTCATGATT |
| 7428F | CTGGGCGCGAAGAATAAAA |
| 8098F | TTTAGTTCAACGGATCCGAGCTAGAA |
| 9611R | GCATTTCTGCTTCAAATTCTTTATCTTTAG |
| 11013F | TTATTTGATTCCAAATTCAGAGCAATG |
| 11024F | CAAATTCAGAGCAATGCCTATTGTACC |
| 11809R | GAAGTAATTGAGCGTGAGAGCCAA |
| 12573R | TTCGTTTCCCTGGGTCACTGG |
| 13857F | TGAAACATATATTGAATTGATCCAAGA |
| 15086F | TCTTGCTGATCAACGATGATTACAACA |
| 15176R | TCCGAAGAGAGATGCAGCCATGTTGAA |
| 15300R | TGTCATTATGTTAATAAAAAATGGC |
| 16012R | CTTCAAGCCAACATAAGAGCATT |
| 16324R | GAAATTATTGTACATCAAATAACATC |
| 16707F | TCTTATTACTACAGAGTCAACTTGAACA |
| 16891R | AAGATTTATAATCCCGACCCAGTAAG |
| 17999R | TATCAAATGCAAGTAGATCGGTT |
| 18514R | TATTAGCAGGAAGGCCACACAAATC |
| 21257F | ATCAGTAAGAGTATTGTTCCGATAGATA |
| 23745F | TGAAATATATACACGAATCGTTTCAG |
| 24047R | ATACCACATTTAGAAGCCCATTTAC |
| 25839F | TCCCAGAGAATTCATTAGAGGAA |
| 27199F | CAATGGTTAATGGTTCAAAATGCTTATGA |
| 27333R | TTGGGGTGGATTACTTAACTTCT |
| 27591R | ACTTCTCGACACCCTTCATAGTATG |
| 28500R | GAAATATCTGTTTGATTGAGACGTTAT |
| 29319R | TCTATGGGATTAAATCCCGAGTTA |
| 30007R | TCTTTCAATTTATTGCCTCTATCTAATG |
| 30617F | ATCCTATCTCGATCCTAATCTTT |
| 31186R | AAGGATTCATAAATAGGAATGGTGCAT |
| 31378F | ATTTGATCCTAGACTCGACGGTAA |
| 31426F | GCCACCAGTCGCTATTAATTTACT |
| 32448R | TTTTCATTTTTCACGGGTCGAGAA |
| 33107R | GTTAAGAAATTGCAGCCTTCCAAATA |
| 41640F | AGTTGACCGAAATGGGCACTAAATAT |
| 42652F | CAACCAATTATGCGCTTCAATATAAT |
| 44098F | AGATGGATATTTGTTGGCCCAAC |
| 44098R | GTTGGGCCAACAAATATCCATCT |
| 44805F | CTCAAATTGCTCCTTTGTGCAGGAA |
| 45619R | AAAGAAGGATCTACCTCTTGCT |
| 46410F | CTCTAGGCTTTTTATTAGTTAATCCT |
| 46756F | TCTTGGAATTGTATTTTAGGATATATATATT |
| 47062F | AGAAAAGCCGGCTATCGGAGTCG |
| 47093R | AGAGCATCGCATTTGTAATGCGATGGT |
| 47223F | GGATTTCAAATCCATATTGCAGTACATT |
| 47442R | GTTTATAAGACTATATTCATTCCAATT |
| 47812R | TAGTTTCGAAATCAAAGCATAGG |
| 48210R | ATTCCAGGGTTTCTCTGAATTTGAA |
| 48324R | TAGAACAGCTTCCATTGAGTCTCTGCA |
| 48912F | TTTGAATGATTCCCAATCTAAGACT |
| 50889F | GTACCAGTCCATAACGATCAAAGT |
| 51375F | ACTAGAAAGTTTCTCTGGGTCTTTTC |
| 51867R | TATACTTTTGTTTCTCGTGAGATAAATT |
| 52167F | AATCCCAATTGGATCCCTTTAAATG |
| 52209F | TTAGGCTTCGGCTCTGAACGATC |
| 52950R | GTGTAAACGAGTTGCTCTACCAACTGA |
| 53371R | ACGAGTGGGTATGTCCGCATAAGCATAA |
| 53397R | GACGAGCTAGGACACGAG |
| 57202F | GGTATTCACGTTTGGCATATGCC |
| 57340R | CCCCTCGTTACGAGCTTGTACACA |
| 57728F | TAGCTTAGTACCGCGGATAGCGAGT |
| 58126R | ACTAGAACCATTACTCCAACTATGAAT |
| 57982F | ATGGAAAAATGGTGGTTTAATTC |
| 59284F | GATCCAATTGTACCACGTAATCCTT |
| 59748R | TTTGCTCCTGTCTTTTAATTCAATATAAA |
| 59748F | TTTATATTGAATTAAAAGACAGGAGCAAA |
| 60984R | CTCTGATTTCTAGATAAAGGACACG |
| 60662F | AGTAGGATTTTTATTAGTTGGA |
| 61592F | GGATTTTTTTGCGATATTTGGATAGTT |
| 62084R | AGAAGAGTCTTTTCTGTAATATCACTCAA |
| 64342F | TAGTTATTACGAATTTAACGGGTC |
| 65416F | AGCAAGGCAATTTCAATTAATATTGA |
| 66175R | GAAATAACTAGTTAGAGTAGGCATGA |
| 66910F | GACGGATTCTATATGTCCAGTTTGCCT |
| 67496R | AGATTTCGCATGGAAAATCCTCCTTC |
| 67732R | TATTCGAAAAGGGAGAAAGGGCA |
| 68009F | TCTATTTACAGCAACGAAATCCT |
| 69081R | TTGTTCGAAAACCCGTAGTTCTAACG |
| 71051F | GTACGTGCAAAAGATGCACATT |
| 71761F | CTGATCCTCGATACTGAGATATACCAT |
| 72416R | GACGCATTTGACCTATATGTGCAA |
| 73201F | CGATAAGTACCAATACGCAAT |
| 74746F | AATATGCTAGACGTGCTCAATTG |
| 75743R | AACTCTAACTTAGTAGTTCTTGTG |
| 79020F | GAGGCAATTCTAATTGATCAATGAA |
| 80192R | TGGGCCTCCGCTGGCACTT |
| 82835F | ATGTCGCGGGCGAATATTTACTCTT |
| 83490R | TGACCCACTTTTGACCCAATTGCTA |
| 84083F | TTGAGCATAATTCTTGATACAATCC |
| 84863R | ATGATTGGCCATACTATTGCTATC |
| **inverted repeat region** | |
| 85109R  (159353F) | TGGGGCTTTCCTGCACTTGGAAG |
| 85527F (158933R) | CTGTACAAGCTTCTTCCAAAGCATA |
| 85824R (158635F) | CGAATTGAAAGCTAAGCAGTGTCTAA |
| 87242F (157217R) | TCAGTGATTCAATTTTCAGTGAAATATC |
| 87787F (159669F) | AAGGATTCTTCCAATGTATAATTTCTGGA |
| 90866F (153593R) | GATGTCTCGTTCAGGTACTTCTTTTC |
| 91599F (152860R) | GGCTAATAGTTTTCATTTCCCATCTC |
| 91943F (152520R) | ACTTGGACTCGGACCCGGAGCT |
| 92314R (152142F) | TCCAAGTGAAATCCCCTAGTATATGAAAG |
| 93017R (151445F) | AACTATGTGATCGACTACATCCT |
| 93046R (151438F) | GAGCCCAAACTATGTGATCGACTACAT |
| 107473F (136987R) | ATTAAAGCGGTACGTGAGCTGGGTT |
| 108472F (135989R) | TTTTTGAGGATACTCCCGGGAACA |
| 108567F (135900R) | GGTTCGAATCCCTCCTC |
| 108921F (135539R) | CTCTCCGGAGAATCGATGACTCCAT |
| 109131R  (135327F) | TGAACGGGTTCGCTTTGACCGTTAAGA |
| 109318F (135141R) | ACC AAT ATG AAC TCG AAG TTT CCT TC |
| 109457R (135007F) | TCTTATGTCAATGACACCAAAGGGATA |
| 109723F (134735R) | AGATAGTCAATTCGGTCGTTGTGGTCG |
| 109887R (143581F) | GAT ATG AAC ATC ATG AGC TGT CCC ATA |
| 110243F (134228R) | GGTATTAGTCTGGATACGGCAAA |
| 110377R  (134078F) | CAAGGTACTTTAATTCTGACACAGAGTACT |
| 110715F (133744R) | AGGATCCGAGTGAATGGAAAGGACAA |
| 110870R (133587F) | TTCATTTCTTACAGCATGTTCGAATCGA |
| 111148F (133310R) | TTGAAACCCTAGACAGAGGATCTCCTT |
| 111317R (133139F) | TTGTCTCTATCGAATTTTTGACTGCAGTT |
| 111522F (132934R) | AGTCTTTTAATCGATGAATTTGCTGAAGA |
| 111681R  (132778F) | TCCATGGGAGGAATTATGGCTGTATC |
| 111979F (132749R) | ACATACTCACAGGCTCTATGCGTGTTC |
| 112040F (132417R) | CTTATATCCGTATTCCCCACTTTTTTTC |
| 112040R (132417F) | GAAAAAAAGTGGGGAATACGGATATAAG |
| 112122F (132334R) | ATGTCTTGTATTAGGTCGGAAATCTCAAT |
| 112450F (132007R) | TCTTATTACGCCAAGAGTCCGAGTGGGA |
| 112537R (131922F) | TCCGTTTCTTCCTGGTTTTAGTAATG |
| 112598R (131858F) | TCAGACGGAAAGGAAATCGTATCATTAGT |
| 112942F (131515R) | ATAATCAGATTATTCATGAATCAGCCAT |
| 113100R (131357F) | TGTTAGGACTAATATTTATCGTTAGAGT |
| 113245F (131214R) | GGATATACACGGATATCCTTCTAGAG |
| 113294F (131161R) | TCTTATTAATAGTCTTTATAGGCCCATGAT |
| 113516R  (130941F) | CTTATACAAGTTAATAACCTGGGTTTGT |
| 113755F (130729R) | GAAATAGAGTCAATCAACATTGTAGGGTTGA |
| 113790F (130671R) | CCAAATTTAATCAAACGGGATTCA |
| 113919R (130537F) | TGTATGTTGTAATTATATGAGTCCTTCTT |
| 114177F (130307R) | AAGCCGATACCTTGGTTATTCCCACAAT |
| 114392R  (130092F) | TGGGCATCTGATGCAAGATCTCTTTGA |
| 114579F (129905R) | TTGCAATTGAAATGGCTGCTGAGTCCT |
| 114775F (129709F) | TACAGGTATACGAACTGGTTCGATAA |
| 115006F (129478R) | GACGGCGTAGAGAATTGAGAATTCTAAG |
| 115079R (129385F) | ACCTTGTTCCCATTGCAAAAT |
| 115174R (129310F) | CTAATCGATAATTGGGCCAAAGAAAGA |
| 115461F (129022R) | ATTGTGTATACCAGATCAAAATACCTC |
| **small single copy region** | |
| 115585F | AAGATAAGAAGAAATGCGGCC |
| 115700F | AGTGTAAGAGGATTGTCCCAATTCAT |
| 116210R | AAACGATACAATCCTTTCTTATTAC |
| 116920R | TCGACTTTTTCCTCTTTTGATAGTAAC |
| 116960R | ACGCAGCTACTATGGTAGCTGCAG |
| 118580F | AACGTGGAATCGAAACTGGATCTATA |
| 119072R | CTACGAATTTTCTATTGGGACCGTCCG |
| 119611F | TTATAAAAATAGCGCCATTGAATTGA |
| 119951R | CGATCGAAAAGGAAATATGATTGAAAAG |
| 120354F | CATTATTATCCGTAGCCCTTTTA |
| 121086F | TAGCTTCTACCTTATCAACTGATAGCGA |
| 121641R | TCCTCGGTATCTCATATGGGTTTCATA |
| 122298R | GGAATAGACGGGCTTTCTATAGGAC |
| 123474F | TAGGCACTCAAAACAAGTACATGT |
| 123964R | GGAATGACTTTGGTAATTTGTACCG |
| 120817F | AGGATTACATAGTTATGGTTCATTT |
| 125983F | GCTTCGGAATTGATCTCGTCCTTTA |
| 128513R | TCAGCACCCATCAATGCATGG |

**Additional file 2 - Taxon-specific primers.**

Primers are named according their position in buckwheat chloroplast genome. For example, for the primers 4080F and 4621R 4080 and 4621 are the starts of the primer sequences on forward and reverse strands, respectively. Primers annealing at the IR region have double name according their position on both IRa and IRb.
