# Supplementary material for: Comparative chloroplast genomics and phylogenetics of Fagopyrum esculentum ssp. ancestrale – A wild ancestor of cultivated buckwheat
Source: BMC Plant Biol. 2008 May 20;8:59. doi: 10.1186/1471-2229-8-59 (PMC2430205; doi:10.1186/1471-2229-8-59)
Supplement: Additional file 4 — Details of the PCR assay of IR expansion, including primer locations and expected amplicon lengths. Table. [file 1471-2229-8-59-S4.doc]

Additional file 4.

| primer name | primer sequence (5’-3’) | location in *Spinacia* cp genome | location in *Fagopyrum* cp genome |
| --- | --- | --- | --- |
| ycf1-SFaln | ATCGATTAGAAGATTTAGCTTGTATGAA | SSC: 121669 – 121696 | IRa: 129265 – 129292 IRb: 115191-115218 |
| ndhF-SFaln | GCTTGTTTTTGGTCCAAAGATGAAAT | SSC: 108740 – 108767 | SSC: 116494 – 116519 |
| rps15-SFaln | AATAGTCTTTTTTGTGCAATTCCAAATG | SSC: 121217 – 121224 | SSC: 128792 - 128819 |

expected amplicon lengths:

rps15-SFaln/ycf1-SFaln: *Spinacia* 445 bp, *Fagopyrum* 500

ycf1-SFaln/ ndhF-SFaln: *Spinacia* no amplification, *Fagopyrum* 1328
