# Supplementary figures and images for: Comparative chloroplast genomics and phylogenetics of Fagopyrum esculentum ssp. ancestrale – A wild ancestor of cultivated buckwheat
Source: BMC Plant Biol. 2008 May 20;8:59. doi: 10.1186/1471-2229-8-59 (PMC2430205; doi:10.1186/1471-2229-8-59)

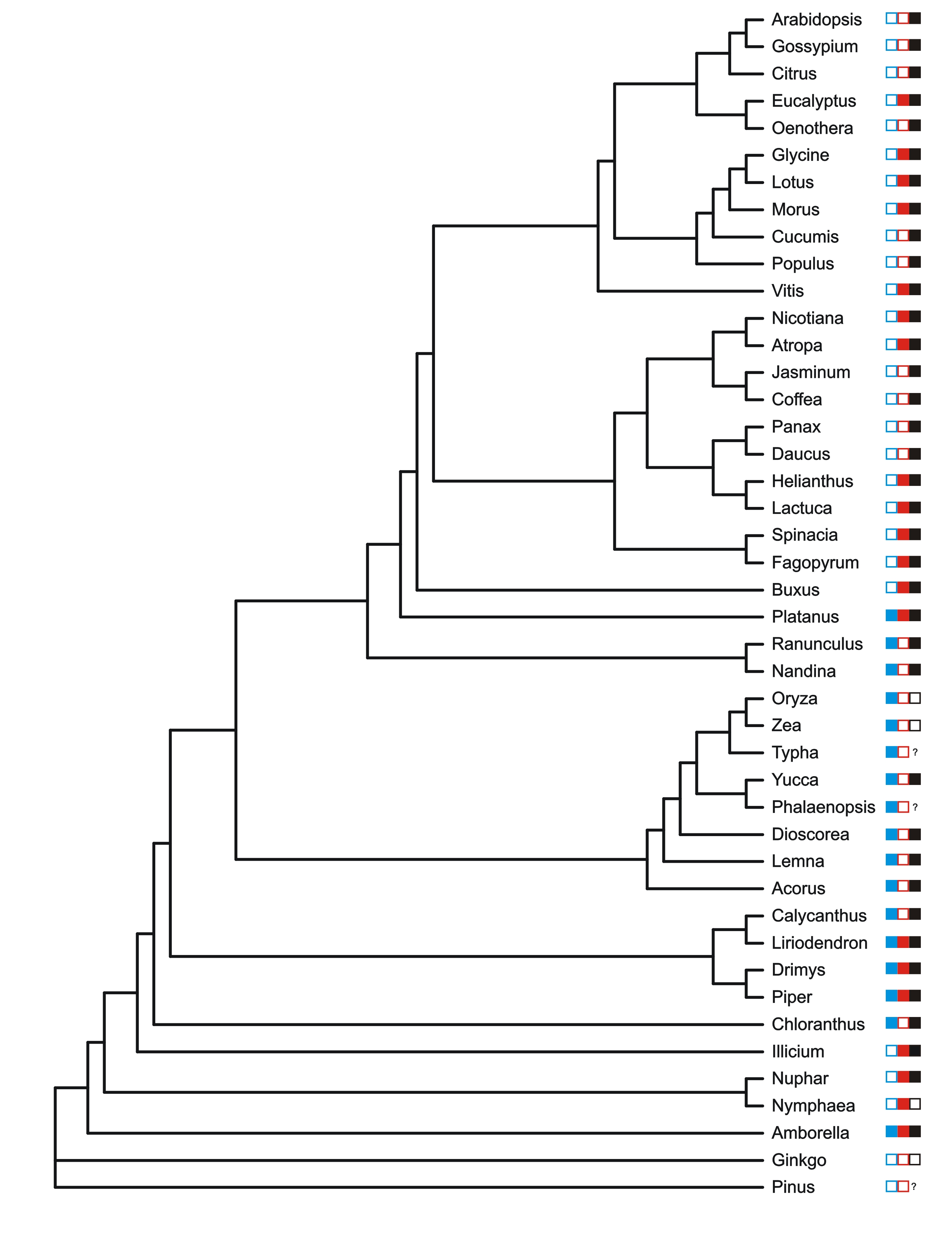

Supplement: Additional file 5 — Distribution of potential RNA editing site in rpl2, psbL and ndhD in angiosperms. Filled squares denote the presence of ACG initiation codon, thin squares – the presence of typical ATG initiation codon. Blue color is for rpl2, red for psbL and black for ndhD. Question marks denote ambiguous character state (due to the loss of gene or the lack of sequence data). Phylogenetic tree is inferred from maximum parsimony analysis of nucleotide data set. [file 1471-2229-8-59-S5.jpeg]
